# Transforming self-experienced vulnerability into professional strength: a dialogical narrative analysis of medical students’ reflective writing

**DOI:** 10.1007/s10459-024-10317-3

**Published:** 2024-02-24

**Authors:** Eivind Alexander Valestrand, Monika Kvernenes, Elizabeth Anne Kinsella, Steinar Hunskaar, Edvin Schei

**Affiliations:** 1 Center for Medical Education, Faculty of Medicine, University of Bergen, Bergen, Norway; 2 Department of Global Public Health and Primary Care, Faculty of Medicine, University of Bergen, Bergen, Norway; 3 Institute of Health Sciences Education, Faculty of Medicine and Health Sciences, McGill University, Montreal, Canada

**Keywords:** Professional identity formation, Medical education, Reflective writing, Dialogical narrative analysis, Transformative learning, Person-centered medicine

## Abstract

Medical students’ efforts to learn person-centered thinking and behavior can fall short due to the dissonance between person-centered clinical ideals and the prevailing epistemological stereotypes of medicine, where physicians’ life events, relations, and emotions seem irrelevant to their professional competence. This paper explores how reflecting on personal life experiences and considering the relevance for one’s future professional practice can inform first-year medical students’ initial explorations of professional identities. In this narrative inquiry, we undertook a dialogical narrative analysis of 68 essays in which first-year medical students reflected on how personal experiences from before medical school may influence them as future doctors. Students wrote the texts at the end of a 6-month course involving 20 patient encounters, introduction to person-centered theory, peer group discussions, and reflective writing. The analysis targeted medical students’ processes of interweaving and delineating personal and professional identities. The analysis yielded four categories. (1) *How medical students told their stories* of illness, suffering, and relational struggles in an interplay with context that provided them with new perspectives on their own experiences. Students formed identities with a person-centered orientation to medical work by: (2) *recognizing and identifying with patients’ vulnerability*, (3) *experiencing the healing function of sharing stories*, and (4) *transforming personal experiences into professional strength*. Innovative approaches to medical education that encourage and support medical students to revisit, reflect on, and reinterpret their emotionally charged life experiences have the potential to shape professional identities in ways that support person-centered orientations to medical work.

## Introduction

Medical students frequently become familiar with medical practice that dehumanizes patients during their education, as they regularly observe patients being “stripped of their uniqueness (stories, personality, culture)” in service to practices that focus on biomedical perspectives over person-centered approaches (Gaufberg et al., 2010, p. 1711). Such dehumanization of patients is unwanted and potentially harmful (Haque & Waytz, 2012), as it precludes the development of a therapeutic alliance and effective healing behaviors on the physician’s part (Cassell, 2012; Kleinman, 2020). Yet, medical students and physicians too often disregard patients’ narratives (Cassell, 2004; Weiner & Schwartz, 2016), emotionally distance themselves from patients’ existential concerns (Schei et al., 2019; Shapiro, 2008; Wilcox et al., 2017), detach from their own emotions (Coulehan & Williams, 2003; Kerasidou & Horn, 2016), and practice medicine in a “courteous but not curious” manner (Agledahl et al., 2011, p. 650). Such dehumanization of self and others may lead physicians to emotional exhaustion, burnout, and distressing emotions like shame (Dyrbye & Shanafelt, 2016; Whelan et al., 2021). A suggested approach to avoid dehumanization in clinical medicine is to help medical students integrate the perspectives and values of person-centered medicine into their professional identities (Santana et al., 2018; Stewart et al., 2013). This may be part of the transformative task professionals undertake as they strive to create coherence between their identity as a person and physician (Moss et al., 2014). While the research on how to support medical students’ professional identity formation is extensive, there is a lack of research into how students inquire into their own emotional lives and prior experiences and how this may be professionally relevant for shaping professional identities that relate to patients in more humanistic ways (Sarraf-Yazdi et al., 2021; Warmington & McColl, 2017). By investigating how medical students in an educational setting reflect on personal experiences that shape their identities, this study contributes to knowledge about how medical education might help students develop professional identities more attuned to person-centered approaches to medicine (Haque & Waytz, 2012; Shapiro, 2013).

Person-centered care recognizes that patients’ health challenges threaten their ability to function as themselves, in their unique context, with their individual goals and purposes (Bansal et al., 2022; Cassell, 2012). The person-centered medical ideal focuses on an interpretivist-constructivist epistemological perspective of what constitutes health and healing (Reeve, 2010). Such an approach recognizes patients’ experiences and perceptions as pertinent to interpretations of illness, suffering, and the healing process. It acknowledges that the quality of the patient-healer relationship influences clinical efficiency and health outcomes (Laine & Davidoff, 1996; Stewart et al., 2013), and that clinicians’ self-awareness is vital for establishing therapeutic alliances between patients and clinicians (Krasner et al., 2009). In medical education, several studies have identified that education about person-centered care needs to be bolstered by theories that clarify the therapeutic rationale for focusing on the personhood of both doctor and patient and the qualities of their professional relationship (Bansal et al., 2022; Haque & Waytz, 2012). As an example, narrative medicine presents a model for physicians to become attentive listeners able to reach deep insights into patients’ lifeworld by utilizing patients’ narratives (Kirmayer et al., 2023; Launer & Wohlmann, 2023; Milota et al., 2019). Coupling theoretical approaches with experiences of shared vulnerability and human connection with patients may help medical students experience patients as “connected to, rather than walled off” from themselves (Shapiro, 2008, p. 8).

In the field of medical education, the professional identity of doctors has traditionally been described as how to “think, act, and feel like a physician” (Cruess et al., 2014; Merton et al., 1957, p. 7). Professional identity is understood to have personal and social dimensions, connecting both to the self and to socialization into the community of physicians (Jarvis-Selinger et al., 2012; Wenger, 1998). In this paper, we focus on how identities form by developing an internalized, ever-evolving story that connects and makes sense of people’s experiences through life (McAdams, 2011). As emotions relate closely to identity formation (Dornan et al., 2015), positive and negative emotions during medical training may impact students’ confidence about their nascent professional identity. They may be driven to act in ways deemed “medical” to cope with feelings of uncertainty, shame, and guilt (Bynum IV & Artino Jr, 2018). Thus, medical students’ inner emotional lives are strongly implicated in forming their professional identities. Medical educators have to consider this and develop new approaches to medical education in response (Lönn et al., 2023; Toufan et al., 2023).

If medical education treats students’ emotional lives with disinterest, detachment, and distancing, it may encourage them to suppress and ignore their own emotions and regard the affective dimensions of patient care as professionally irrelevant (Shapiro, 2011). In contrast, awareness and acknowledgment of the function of emotions may increase students’ resilience and help them see suffering, grief, pain, resilience, and healing as potential sources of strength (Shapiro, 2008). This theme is salient in Greek mythology, where the centaur *Chiron*, pierced by an arrow, was the ‘wounded healer’ who, through his suffering, understood how to ease others’ pain (Graves, 2017; Jung, 1951). Accepting vulnerability and suffering as existential necessities and fundamental human experiences can provide life with meaning (Binder, 2022; Frankl, 1985), and potentially help physicians use their wounds as resources for connecting with patients (Daneault, 2008).

When medical students partake in person-centered learning activities, they may experience the interpretivist epistemological knowledge paradigm, rooted in recognition of how people interpret meaningful experiences and construct knowledge through stories, conflicting with the biomedical, positivist epistemology of medicine, which focuses on diagnosis and management of disease (Bansal et al., 2022). This tension may create identity dissonance as students struggle to integrate “a different world-view, different values and emotional orientations” (Monrouxe, 2010, p. 42). There is a risk that students resolve the dissonance by letting the diagnosis-centered perspective predominate, concluding that person-centered medicine is neither important nor helpful for them as doctors (Bansal et al., 2022). A perspective transformation can occur when educators interweave person-centered theory with meaningful experiences while providing affective support and small group sensemaking that enable students to integrate person-centered beliefs, values, and attitudes into their professional identities (Bansal et al., 2022; Dornan et al., 2014; Schei et al., 2019). Meaningful experiences that have the potential to shape medical students’ professional identities include encounters with patients, the influence of role models, and prior life events from before medical studies (Wong & Trollope‐Kumar, 2014).

The aim of this study is to explore how written reflection on personal life experiences and their relevance for future professional practice can inform first-year medical students’ initial explorations of professional identities. The material is based on reflective essays written after exposure to patient encounters, person-centered theory, and role modeling in an educational setting.

## Methods

### Context

The data for this study are reflective essays written by first-year medical students at the end of a 6-month course called Patient Contact (PASKON). The course aimed to foster transformative learning (Slavich & Zimbardo, 2012), enabling students to embody a person-centered perspective on the goals and practice of medicine as part of their professional identity formation. In the course, students were exposed to teaching and reflection about person-centered medicine, humanistic values, and communication skills. They encountered patients in small groups and engaged in reflective writing. Course sessions were guided by eight physicians from various clinical specialties who taught in pairs and aspired to role-model person-centered communication, student-centered reflective dialogue, and relational self-awareness. They aspired to normalize emotions as necessary, helpful, and integral to medical work and professionalism (Schei et al., 2019). In addition, students received support from senior student supervisors trained by course facilitators. The supervisors had all previously taken the course, they met and helped the students before the patient encounter, and provided feedback on their reflective writings afterward.

An introductory phase of the course involved live demonstrations of physician–patient communication, reflection in groups with peers, and an introduction to person-centered theory (Cassell, 2004, 2012). Demonstrations of course educators communication with patients were followed by student volunteers speaking with patients in front of the class while getting guidance from course educators and peer students. Students in small groups were encouraged within their personal comfort levels to share personal stories of illness and healthcare encounters, discuss their experiences with shame, and reflect upon the goals of medicine. As an introduction to person-centered theory, students were introduced to Cassell’s (2012) definition of a sick person as one “who cannot achieve his or her purposes and goals because of impairments of function that are believed to be in the domain of medicine” (p. xvi). This definition served as a basis for introducing physicianship and person-centered medicine, encouraging students to pay attention to a person’s personal goals, motivations, roles, and functions, and how these are impacted by, and shape, a patient’s everyday life, diagnosis, and treatment. Teachers also addressed theoretical concerns that some students might experience emotional distress during a presentation of patients’ narratives. This was normalized, and students were explicitly invited to contact teachers at any point if they experienced emotional triggering. Student supervisors were also told to speak with the course leaders if any reflective writings raised concerns about students’ well-being.

In the next phase, students in 40 small groups of four to six visited a patient with a chronic health condition at home. The task was to explore, through dialogue, “how a person’s life is affected by illness”. A few days following the visit, students and patients presented together in class. For the presentations, the class was divided in two, meaning that all students over a few months were introduced to 20 patients’ stories. For instance, patients shared the life crisis they had when they were diagnosed with diabetes in their teenage years, how it felt to gradually become blind and withdraw socially, or their fear of dying from cancer and not being able to see their children grow up. During the presentations, students sat together with the patient in a semi-circle to enhance the patient’s feeling of being supported and safe. The non-presenting students were asked to sit near the front of the classroom, remove distracting elements (i.e. cell phones), and practice listening to the patient’s story. The patient was invited as an ‘experience expert’ into a dialogue about the patient’s lifeworld, drawing on the principles of respect and curiosity (Lefkowitz et al., 2022). At the end of the presentation, the students in the small group were asked to share what they learned from meeting the patient and hearing about their illness experience. Making students’ learning outcomes explicit can help patients feel that their participation in an educational program was meaningful (Lauckner et al., 2012). Students wrote two reflective essays about the patient encounter immediately after the patient home visit. In these essays, students were first asked to reflect on how it was to be them and then on how they believed it was to be the patient during their encounter.

### Narrative methodology

The team was crucial in planning the study, ensuring we had a robust theoretical and methodological framework. This study draws on narrative inquiry methodology within a socio-constructivism research paradigm (Frank, 2010). Narrative inquiry attends to the “relational aspects of living and telling stories, of context and person, of researcher and research participant” (Clandinin et al., 2017, p. 91). In medical education, narrative methods have been used to research professional identity formation (Monrouxe, 2010). Narratives reveal identities as people “convey to themselves and to others who they are now, how they came to be, and where they think their lives may be going in the future” (McAdams & McLean, 2013, p. 233).

We chose dialogical narrative analysis (DNA) (Frank, 2010, 2012) due to its suitability for exploring identity while recognizing that a single story expresses the multiple voices it has been influenced by (Gubrium & Holstein, 2009). It was essential to acknowledge the reflective essays as elicited by an assignment in a course; and to make explicit that they were designed in a context, for an audience, anticipating a response (Frank, 2010). The principles of dialogism underpinning DNA build on Mikhail Bakhtin’s work. He saw identity as the ideological becoming of self (Bakhtin, 2010), which unfolds through dialogue with some other, “whether that other be another person, other parts of the self, or the individual’s society or culture” (Josselson, 2011, p. 41). The construed identity is seen as never fixed, as stories can be continuously revised, making our identities dynamic, ever-evolving, and unfinalizable (Holquist, 2003). Narratives can call individuals into groups with common identities when shared stories resonate with a person’s inner stories (Frank, 2010).

### Data collection

In the later phase of the course, students were presented with three options for a third reflective essay. In the first option, students were invited to “write a reflective text about yourself and how experience has shaped you. Take as a starting point something you have experienced in your life or a relationship you have been in, and reflect on how you think that experience can contribute to you becoming a better (or worse) doctor than you would otherwise”. See Table 1 for a description of the assignment. The essays of students who chose this option constituted the data for the current study. The two other options (not part of this study) were for students to write about (a) what they learned throughout the course or (b) the importance of trust in medicine. The intention was to offer students the freedom to choose a reflective essay question requesting personal disclosure within their comfort zone.

**Table 1:** Instructions for the reflective essay assignment analyzed in this study

Reflective essay assignment no. 3 Write a reflective text of 800–1500 words about yourself and how experience has shaped you. Take as a starting point something you have experienced in your life, or a relationship you have been in, and reflect on how you think that experience can contribute to you becoming a better (or worse) doctor than you would otherwise be. Link your text to at least three concepts from chapter 8 in “Listen”*, and explain why you think the concepts shed light on what you are writing about	
Here are some examples of experiences that can affect a future doctor’s role:	
You are multicultural	You were very sick as a child
Mother died	Parents were divorced
Dad was imprisoned	Sister got anorexia
The neighbor was a wonderful doctor	Grandma was good at telling stories
A movie	A book
A teacher	Etc., etc., etc.
* «Listen. Physician role and communication» is a Norwegian textbook (Schei, 2015) introducing theory and multiple examples of physicians’ professional experiences, person-centered medicine, clinical communication skills, psychotherapy, and medical philosophy	

All the reflective essays had a formative purpose, in which students were told that failing was not an option. However, they needed to engage in the assignment, or they might be asked to rewrite and improve on their initial writings. The intent of the assignment was made explicit; its function was to help them develop as future physicians, not to grade or fail students. The essays were read, and written feedback given, by the senior student supervisors. At the end of the course, a written exam assessed students in PASKON and other subjects from the semester.

In the classes of 2018 and 2019, 114 of 333 students chose to write their third course essay on how they envision that a personal experience or relationship from before medical school may influence them as future doctors. In 2020, after students had completed all their coursework, a researcher (ES) contacted students by email to invite consent to include their essays in this study. Written consent was obtained from 68 of 114 students. Among these were 49 females and 19 males, corresponding to the gender balance in these classes. There was a mix of cultural backgrounds, age, and social class among respondents. A researcher (EAV) anonymized the essays of consenting participants by assigning numbers before analysis.

### Data analysis

After an initial reading of the dataset, we decided on DNA as a productive method for examining identity formation within a contextualized narrative dataset (Frank, 2010). Before analysis, the research team discussed the context in which the reflective essays were written. Key considerations included what could influence which story students decided to write (e.g. experienced psychological safety in the course), who the students knew would read their stories (e.g. senior student supervisor providing feedback), and the course’s explicit and implicit experiential and theoretical components (e.g. patient home visit, textbook, and educators role-modelling). Then, two researchers (EAV, ES) closely read the 68 texts and discussed them in detail together. We applied Bamberg’s stepwise interpretation of content and context by discussing how the student told their story, how the story was constructed for the reader, and how students displayed notions of selves (Bamberg, 1997; Konopasky et al., 2021). The interpretation first focused on each individual essay, before we discussed our interpretations across the essays. We engaged specifically in Frank’s proposed ‘crucial question’ to inquire into how students held their own in the act of storytelling (Frank, 2012). By holding one’s own, we mean to support and delineate one’s identity through storytelling. The question allowed us to explore how medical students form identities when narrating their past and possible future in the present. A table of the data material was aggregated, with short summaries of each narrative, students’ use of course terms and theoretical concepts, striking quotes, our comments, and initial notes regarding plot, characters, setting, and topic for analyses. This helped us juxtaposition the stories with one another to develop a deeper understanding of meaning.

We discussed preliminary findings with the entire research team. The team engaged in dialogue about the emerging findings and iteratively worked with the material, including conversations regarding presenting the findings and the implications for medical education. As we explored the theoretical literature, we became more sensitive to nuances of meaning, making it necessary to revisit the data continuously during the writing process.

### Reflexivity

We organized our work to optimize the interplay of the different experiences and fields of expertise within the research team, representing a variety of competencies and lenses for observation and analysis. In addition to medical (EAV, SH, ES), or educational (EAK, MK) training, the team had experience in teaching and supporting medical students, health professions education, qualitative methodology, and philosophical inquiry. The team brought research interests in professional identity formation, person-centered medicine, and reflective writing.

The research team offered a balance of insider and outsider views on the context of the students’ reflective writing. The authors most hands-on with the data (EAV, ES) have both taken part in creating and teaching the course in question. In addition, EAV has experience as a student supervisor and patient in the course, while ES has written the course textbook (Schei, 2015). The other researchers (MK, EAK, SH) have been neither teachers nor participants in the course and could challenge data interpretations by applying their pedagogical, methodological, clinical, and theoretical perspectives.

### Ethical considerations

Throughout the research project, we were mindful of potential ethical considerations and reflected on these at each step of the research process. We worked to maintain a high ethical standard to ensure the safety and care of all participants, including patients in the course and the study informants. We were aware of the sensitivity of the vulnerable experiences that were being shared (Lee & Renzetti, 1990), and how telling one’s story can be an emotionally complex endeavor that requires support (Roebotham et al., 2018). During data collection, all information came from ES, the primary teacher in the course and a person with whom students had an established relationship. Presenting the results as narrative dimensions was an ethically informed decision, ensuring students’ anonymity while highlighting and respecting that vulnerable life experiences are the norm rather than the exception.

Patients in the course were invited to participate based on their physician and themselves deeming them ready to share their stories while having the perceived resilience to cope with the experience. Students were instructed in approaches to support patients before and during the class, with course educators facilitating a caring dialogue in the classroom. For instance, patients were explicitly told they did not need to answer questions or share anything they felt uncomfortable with. If there were grounds for concern about the patient’s emotional state following the presentation, the course leader contacted the patient. Most patients wished to return in future years and have participated several times in past years.

For the presentation of the results, the students’ reflective writing was assigned randomized students’ numbers (e.g. Student 1). The class year is not indicated to further protect students’ anonymity.

## Results

The analysis yielded four categories. We first present (1) *how medical students told their stories* of illness, suffering, and relational struggles in an interplay with context. This is followed by insights about how students formed identities through narration. These categories highlight learning processes that support professional identity formation congruent with a person-centered orientation to medical work: (2) *recognizing and identifying with patients’ vulnerability*, (3) *experiencing the healing function of sharing*, and (4) *transforming personal experiences into professional strength*.

1. How medical students told their stories

The students’ personal stories were diverse, containing significant thematic breadth. They told of struggling with body image and eating disorders; of being bullied with subsequent depression and self-harm; of following a mother through cancer and holding her hand as she drew her last breath. Others told stories of being there for a friend who is depressed and thinking of suicide; of having a summer job at a nursing home and becoming attached to older adults; of being a top-level international athlete who meets new cultures and must take responsibility from an early age; or, of being a child war refugee. While the plots differed, the stories' nature tended to involve complex experiences connected to feelings of vulnerability.

*“I have experienced abuse, I have felt transparent, I have intended to take my life, I am multicultural, I feel alone sometimes, I deny my feelings, I go into therapy, I am happy and full of life, I am encouraging with everyone, I want to be seen, I am a human being.”*—Student 4

That students were willing to narrate stories like this suggests that the course successfully created a psychologically safe space. The course was designed to stimulate students to display their own emotional vulnerability by explicitly addressing emotions and personal stories and by exposing students to personal narratives of being human. The diversity of stories suggests that students were willing to tell authentic stories about their experiences, focused on emotionally demanding and challenging events with personal thoughts and feelings at the center.

A number of students noted that being introduced to new concepts, words, theory, and the narratives of others supported them in finding words as they reflected upon their own stories. One student noted how the course materials allowed them to reflect on an experience’s deeper meaning and verbalize it.

*“Before I started writing this essay, I did not know that I had experienced something special in my life that has left a deep mark on me, that will shape me as a doctor. … I want it to be a deeper meaning. The course and the textbook have helped me verbalize this meaning”.*—Student 43

A few students had not integrated theoretical perspectives from the course material into their reflective writing but rather appeared to add theory ‘on top’ at the end of the essay. These students still displayed reflexivity and depth in their writing. However, they seemed to struggle with capturing in writing how applying theory might have helped them reach new insights from their experiences.

As students composed their narratives, some noted the formative influence of experience on their identity. Students discussed how these life events shaped their conscious ways of thinking. Some also described the unconscious and embodied impacts of experience, indicating how they saw their story as part of themselves:

*“Things we have felt in the body, both in the physical and emotional sense, are not transient events that lose their impact after they are experienced; our experiences become part of our being.”*—Student 55

In the following, we will present the three dimensions identified in which students ‘hold their own’, meaning how they narrated to create coherence between their personal and emerging professional identities.

### How medical students formed identities through narration

2. Recognizing and identifying with patients’ vulnerability

Students told their personal stories in ways that emphasized a relation with patients. One student’s story is presented as an exemplar. The student described the time when her mother died as challenging and heartbreaking but also colorful and filled with love. While reflecting on the story, she considered how, as a future physician, she must perceive the needs and desires of patients. She connected her own needs and those of her family when they suffered with the prospect of alleviating the suffering of future patients.

*“When I now, for the first time with my medical student glasses on, reflect on this time, there is one thing in particular I keep coming back to: the importance of unimportant things. By unimportant things I mean circumstances that, compared to the amount and types of symptoms and chance of survival, seem very modest. Especially for a doctor, who can quickly get caught up in the urge to fight and overcome the disease. For the patient, on the other hand, these unimportant things can be absolutely essential for the quality of life.”*—Student 14

She reasoned that when a sick person feels that they can pursue some of their personal goals and desires, such as "*being with friends, going to work, shaving your legs, and maintaining your hobbies” (Student 14),* the disease’s ability to take control over life is reduced. Reflecting on this experience, she realizes that her own experience of vulnerability offers a window for recognizing how to help others live with severe illness.

A number of students who identified with patients’ vulnerability wrote about the need to stay with and acknowledge that vulnerability and not distance themselves from it, even though it might be uncomfortable to be close to a person’s suffering. Some became aware that they tried to protect themselves through distancing:

*“So I was most concerned with myself and the feelings I had, and protected myself from strong impressions, hurt and powerful feelings and not least the rejection.”*—Student 11

Recognizing their shared vulnerability helped students see patients as individual, emotional beings like themselves. Some of the students wrote an imagined dialogue with patients in which this became explicit. In the excerpt below, a student writes about recognizing the common humanness while honoring the individual difference:

*“But who are you? It is easy to think that you are like me and that my thoughts, opinions, and experiences can be directly transferred to you, but this is of course not the case. In many ways we are equal, because we are both human, but as individuals we are very different.”* —Student 19

3. Experiencing the healing function of sharing

Students described how divulging their secret stories of suffering provided relief and strength but also took courage. Many drew connections to how it may be healing for patients to share stories, thoughts, and feelings during the clinical encounter. Some students wrote of the obligation as future doctors to stay with what is difficult and emotional:

*“It can seem big and scary to go into the difficult topics, sometimes impossible, but you have to dare to try if you are to have the opportunity to actually help someone.”*—Student 9

Students reflected on how to help others share their inner thoughts and feelings, variously writing about listening, empathy, non-judgment, and acceptance:

*“I imagine that we all have a life task in practicing listening. To listen, completely uninhibited, to what the person in front of you has to say, without having thought about what you want to answer, creating a space where the patient can take the time they need to formulate themselves without fear of being judged, being shamed or rejected.”*—Student 38

The importance of creating a space of trust and being present with patients to allow for the sharing of stories was also highlighted:

*“When you first meet a new person, it’s like stepping into the unknown. So much can go wrong, but if you succeed, you have the chance to change a part of someone else’s life story in a positive way.”*—Student 27

Many students described feelings of hesitancy as they wrote about their experiences and vulnerabilities, wondering whether it was:

*“ (…) wise to open up about the most vulnerable. All the bad feelings and the eternal sorrow that very few know about. It feels unsafe, but at the same time necessary. I have actually realized that if I’m going to manage to complete my medical studies, I’m forced to come to grips with what I have suppressed in my subconscious until now.”*—Student 51

Commenting on the decision to choose the assignment involving a life story, many students indicated that they understood better, through writing, why it was important for them to do so. Underscoring the therapeutic effect of writing, some discussed how it helped them process past experiences or make sense of them in ways that might be beneficial for them as future doctors:

*“This text is actually a right step in the direction of working with it and becoming familiar with it, because I have not been good at talking openly about the eating disorder I struggled with. What I do now may therefore be important so that I can better talk about shame with my patients, and let them have an outlet for it in my possible future doctor’s office.”*—Student 47

4. Transforming personal experiences into professional strength

For many, what had hitherto been a never-told story of weakness, suffering, and emotional vulnerability, a potential source of shame and insecurity, became a proud story of having lived in ways that could yield some of the strength, wisdom, and understanding of others that is required in a physician:

*“My experiences have led me to feel ashamed of not feeling like a full-fledged human being, but a broken, injured, and ugly human being who no-one will ever think is good enough. (…) My survival tactic was to become a good listener and mind-reader, no one should feel that I did not see them, and no one should have to say more than they wanted. I had to grasp quickly what they felt, thought, and needed, without them having to say anything. In a doctor-patient situation, I see that this can be a strength and something I can use in the future.”*—Student 38

For many students with emotionally laden stories, storytelling had an empowering effect, transforming their view of themselves as future clinicians. Many expressed an emerging insight that having normal human emotions and common experiences of difficulties and resilience were essential assets for doctors:

*“None of my previous problems are that unique, there are plenty of people who have both felt and experienced much of the same as I, and far, far worse things. But I still feel better equipped now, than I otherwise would have been, to be able to understand my fellow human beings' challenges and understand them. My interest in people's history and background, as well as my patience with others, makes me well-equipped for meeting patients.”*—Student 10

## Discussion

This study shows how first-year medical students can familiarize themselves with the patient perspective and understand its medical relevance by exploring their own emotionally charged life experiences. Having listened to patients’ stories and been introduced to person-centered medical theory, students’ reflective examination of their own life experiences revealed three ways of “holding their own” in narration. In terms of identity formation, the process showed the potential for connecting students more deeply to patients’ vulnerability, fostering insights about the healing power of sharing stories, and helping students transform difficult personal experiences into professional strength. Their capabilities as future doctors may improve as they appreciate the impact of situated personal experiences on health and healing and learn to use relational skills in medicine.

For the research team members, it was an awakening to become aware of the powerful lived experiences of medical students. The stories were richer and contained more suffering than we anticipated, and aligned with Henri Nouwen’s contention that “[w]e are all wounded people, whether physically, emotionally, mentally, or spiritually” (Nouwen, 1979, p. 199). What appears to vary, however, is the capacity to use experiences of emotional vulnerability as a source of professional strength in care for others.

Our study suggests that emotionally salient life events in medical students are relatively common. Existing literature gives reasons to fear that vulnerable experiences may trigger unhealthy defense mechanisms, such as distancing from the patient and feelings of shame, if left unresolved (Bynum IV & Artino Jr, 2018; Schei et al., 2019; Shapiro, 2011). Our findings highlight how individual experiences of suffering may harbor a potential for becoming sensitive to our ‘wounded humanity’, along with that of patients (Johna & Rahman, 2011). Many students appeared to be open to the connection between emotions and bodily symptoms and to accept that to relieve the suffering of others, physicians need to familiarize themselves with what may be vulnerable, emotional, and difficult. This study shows how enabling an agentive act of storytelling can help students make sense of their vulnerable experiences, reach deeper insights about existential dimensions of being human, and see person-centered medicine as a tool for developing therapeutic alliances (Cassell, 2012; Shapiro, 2008). We contend that reflective educational approaches that foster opportunities for students to examine their own vulnerable experiences have the potential to connect students to what Jung (1951) termed the ‘wounded healer’: A wounded healer accepts their vulnerability and the existential suffering they are subject to, and has the potential to integrate this knowledge into a perspective that they can use to connect with and care for the patient (Merchant, 2012; Zerubavel & Wright, 2012).

Developing a sound professional identity can be difficult if students experience dissonance between a personal, emotional identity and a perceived non-emotional professional ideal (Monrouxe, 2010). In the current setting, such dissonance was potentially mitigated, as both the theoretical curriculum, the role models, and the reflective writing exercises helped students understand how personal life stories can permeate a clinician’s tacit perception of what it means to be a doctor. In the course, we do not present identity as a way of being that students are asked to adopt uncritically; they are instead encouraged to explore with guidance how to become a doctor capable of being both genuinely personal and professional, with a recognition that identity formation is dynamic, ever-evolving, and unfinalizable, rather than a final product to be achieved (Holquist, 2003). The reflective writing made use of “our narrative gift that gives us the power to make sense of things when they don’t” (Bruner, 2003, p. 28). Students created narratives that helped them see the professional value of their experienced vulnerability and suffering, thus potentially transforming personal weakness into professional strength and dissonance into congruity between multiple identities. Though these students are at the beginning of their careers, they experience emerging professional identities in which physicians and patients are perceived as connected, emotional beings, sometimes vulnerable and imperfect, yet respectable and capable. Hopefully, this will help the students establish proximity to patients while being able to contain the suffering of their patients and themselves (Shapiro, 2008).

Strict ground rules about privacy and confidentiality and teachers’ and senior student supervisors’ honesty about their own vulnerability were elements aimed at constituting safety in the course. Experiencing role-modeling of openness and emotional self-awareness helped students develop attitudes they may apply as doctors to create trust in the physician–patient relationship (Passi et al., 2013). Theory highlighted the role of emotion in healthy functioning, illness, healing, and growth (Shapiro, 2008). Reflective writing for a senior peer reader has the potential to allow students to perceive the healing power of storytelling within therapeutic relationships (Charon, 2008). By experiencing this themselves, students may realize that patients’ vulnerable stories will only be shared in psychologically safe spaces (Edmondson, 1999), where it is clear that emotional frankness and displays of distress will not elicit judgment, criticism, or rejection (Shapiro, 2013).

**Fig. 1:**
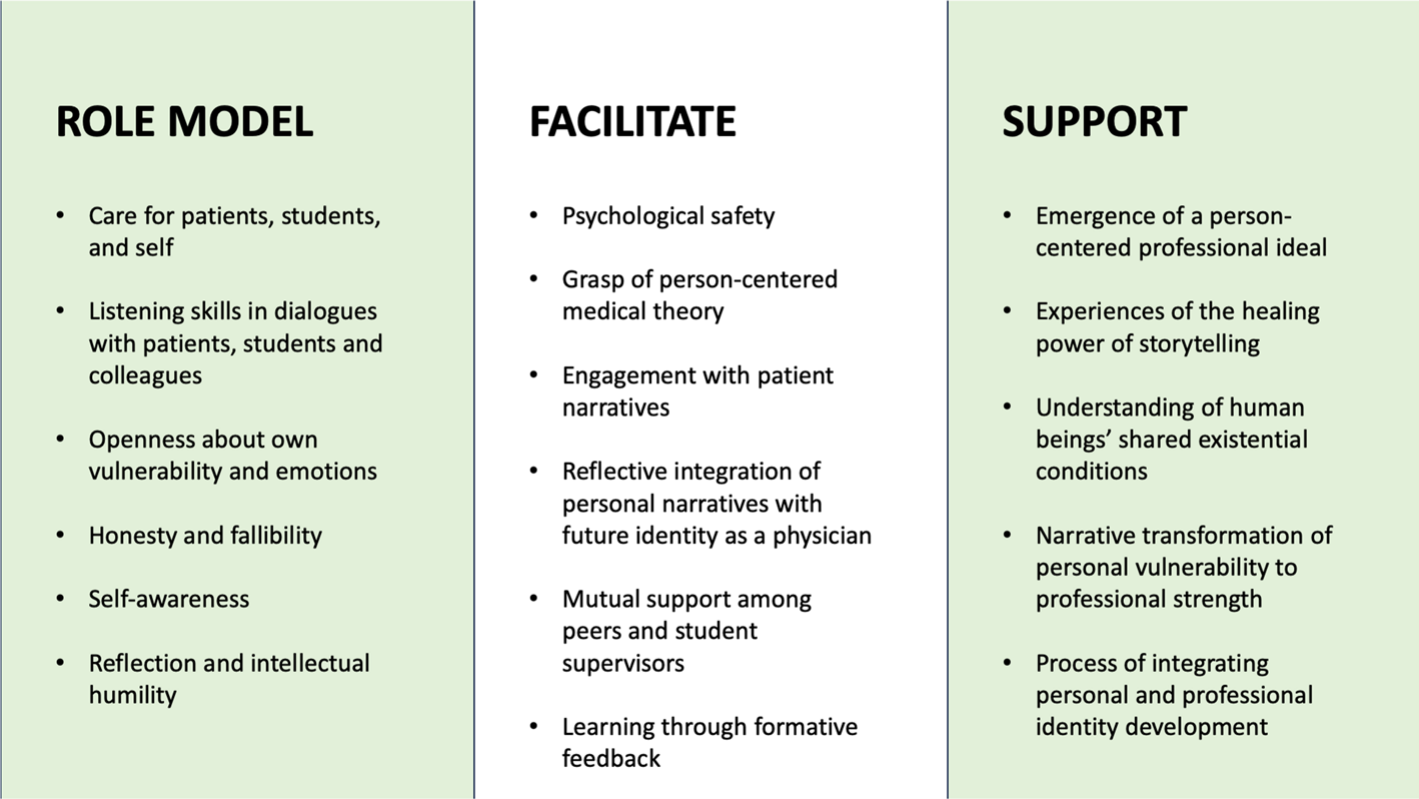
Proposed educational elements to help students integrate person-centered perspectives

Some students may have felt incapable, unsafe, or unprepared to reflect on themselves critically, and may therefore have chosen to respond to a different task for their third reflective essay. The other tasks may also have felt more interesting or less challenging. For those who decided to write about their personal experiences, elements in the course impacted why and how they decided to tell their story. They are writing to a trusted respondent in a context where person-centered medicine is advocated, and emotions, reflexivity, and diversity are appreciated. The likelihood of these stories and reflections being authentic, with students engaging in actual identity work facilitated by the educational intervention, is underpinned by the emotional honesty and vulnerability portrayed in the studied narratives, the personal dimension displayed, that the essays were not constructed as part of an assessment, and that the students were free to choose a less personal assignment (De la Croix & Veen, 2018). If, for instance, the students had been asked to write for the course teachers rather than student peer supervisors, the dialogue would have changed, and the stories would have been different. Others wanting to implement such educational interventions must consider how these elements play together to enable transformative learning (Slavich & Zimbardo, 2012). Drawing on a synthesis of the scholarly literature included in the course, our practice-based experience, and the present study, we propose educational elements outlined in Fig. 1 that can help medical students’ to integrate person-centered perspectives. This includes guidance on what educators can role model, educational activities they can facilitate, and the direction of support required.

This study suggests that the shaping of person-centered physicians of the future may be facilitated by allowing medical students to revisit, reflect on, and reinterpret past experiences and feelings while imagining their future selves as physicians. Reflecting back on emotionally salient life events may help medical students form professional identities in which their personal vulnerability becomes a tool for connecting with patients, helping them be present and listen to the other’s stories on a deeper level. Medical students’ personal reflections need to be framed in psychologically safe surroundings with personal support and a clear rationale for reflective exercises (Bansal et al., 2022; Edmondson, 1999; Lim et al., 2023). In addition, coupling students’ personal reflection with person-centered medical theory can deepen reflections about the nature of health and sickness, the doctor-patient relationship, and students’ visions of themselves as future physicians. For professionals who integrate an individualized and humanized view of patients into their narrative understanding of what it means to be a person, the urge to maintain a coherent understanding of themselves can provide resistance against forces that may lead physicians away from a person-centered practice of medicine (Wilt et al., 2019).

### Limitations and further research

It is important to acknowledge the limitations of this study. First, the short stories analyzed in this study give insight into only some aspects of identity while others remain hidden. The future trajectory and continuing development of these first-year medical students are uncertain and could be a focus of future research. Perhaps students are more receptive to this type of reflection at this early stage of training. Other studies could explore how students already socialized into biomedical ways of thinking and being can be helped to integrate person-centered perspectives. Second, we have yet to learn about the perspectives of students who chose other writing tasks. It may be that they cannot access their “inner library” of life stories (Frank, 2010) or chose a different task for other reasons. Further research could explore how narrative curricular interventions might be utilized across a broader group of medical students to explore reflection on personal experience and the implications for developing professional identities. Third, some reflective writings may become an ‘act’ intended to satisfy supervisors rather than a genuine reflection in themselves (Liao et al., 2023; Monrouxe et al., 2011). Therefore, the findings must be interpreted with recognition that the writing took place within a course context and that students may have wished to satisfy the course instructors or senior students reading their work. It is important to note, however, that emphasis was on formative rather than summative assessment and that no grades were assigned to the essays, which may have fueled more safety for authentic participation (Watling & Ginsburg, 2019). The efforts to ensure students’ psychological safety, the freedom to choose from a range of assignments beyond the more personal one, and the invitation for diverse stories were other elements in the course intended to help mitigate against false representations and support authentic reflection (De la Croix & Veen, 2018).

## Conclusions

This study shows that medical students’ personal experiences from before medical school harbor a rich potential for informing a person-centered perception of medicine and strengthening the affective dimension of medical education and professional identity formation. The personal dimension is crucial for physicians’ clinical capabilities and may foster a capacity for connecting with patients and support healing. Medical educators need to be aware of the emotional power of students’ personal stories and their potential to impact, negatively or positively, on students’ future professional identity, the embodiment of professional ideals, and the physician–patient relationship. If students are adequately supported and encouraged during their education, they may acknowledge, share, reflect on, and reinterpret their personal stories of illness, suffering, and loss. Then, students can embark on a process of transforming themselves into wounded healers—somewhat stronger and somewhat wiser.

## References

[CR1] Agledahl, K. M., Gulbrandsen, P., Førde, R., & Wifstad, Å. (2011). Courteous but not curious: how doctors’ politeness masks their existential neglect. A qualitative study of video-recorded patient consultations. *Journal of Medical Ethics,**37*(11), 650–654. 10.1136/jme.2010.041988 21610269 10.1136/jme.2010.041988 PMC3198010

[CR2] Bakhtin, M. M. (2010). *The dialogic imagination: Four essays*. University of Texas Press.

[CR3] Bamberg, M. G. (1997). Positioning between structure and performance. *Journal of Narrative and Life History,**7*(1–4), 335–342. 10.1075/jnlh.7.42pos

[CR4] Bansal, A., Greenley, S., Mitchell, C., Park, S., Shearn, K., & Reeve, J. (2022). Optimising planned medical education strategies to develop learners’ person-centredness: A realist review. *Medical Education,**56*(5), 489–503. 10.1111/medu.14707 34842290 10.1111/medu.14707 PMC9306905

[CR5] Binder, P.-E. (2022). Suffering a healthy life—On the existential dimension of health. *Frontiers in Psychology,**13*, 803792. 10.3389/fpsyg.2022.803792 35153958 10.3389/fpsyg.2022.803792 PMC8830493

[CR6] Bruner, J. S. (2003). *Making stories: Law, literature, life*. Harvard University Press.

[CR7] Bynum, W. E., IV., & Artino, A. R., Jr. (2018). Who am I, and who do I strive to be? Applying a theory of self-conscious emotions to medical education. *Academic Medicine,**93*(6), 874–880. 10.1097/acm.0000000000001970 29068821 10.1097/ACM.0000000000001970

[CR8] Cassell, E. J. (2004). *The nature of suffering and the goals of medicine* (2nd ed.). Oxford University Press.

[CR9] Cassell, E. J. (2012). *The Nature of Healing: The Modern Practice of Medicine*. Oxford University Press.

[CR10] Charon, R. (2008). *Narrative medicine: Honoring the stories of illness*. Oxford University Press.

[CR11] Clandinin, D. J., Cave, M. T., & Berendonk, C. (2017). Narrative inquiry: A relational research methodology for medical education. *Medical Education,**51*(1), 89–96. 10.1111/medu.13136 27807868 10.1111/medu.13136

[CR12] Coulehan, J., & Williams, P. C. (2003). Conflicting professional values in medical education. *Cambridge Quarterly of Healthcare Ethics,**12*(1), 7–20. 10.1017/s0963180103121032 12625198 10.1017/s0963180103121032

[CR13] Cruess, R. L., Cruess, S. R., Boudreau, J. D., Snell, L., & Steinert, Y. (2014). Reframing medical education to support professional identity formation. *Academic Medicine,**89*(11), 1446–1451. 10.1097/acm.0000000000000427 25054423 10.1097/ACM.0000000000000427

[CR14] Daneault, S. (2008). The wounded healer: Can this idea be of use to family physicians? *Canadian Family Physician,**54*(9), 1218–1219. 18791082 PMC2553448

[CR15] De la Croix, A., & Veen, M. (2018). The reflective zombie: Problematizing the conceptual framework of reflection in medical education. *Perspectives on Medical Education,**7*, 394–400. 10.1007/s40037-018-0479-9 30353284 10.1007/s40037-018-0479-9 PMC6283773

[CR16] Dornan, T., Pearson, E., Carson, P., Helmich, E., & Bundy, C. (2015). Emotions and identity in the figured world of becoming a doctor. *Medical Education,**49*(2), 174–185. 10.1111/medu.12587 25626748 10.1111/medu.12587

[CR17] Dornan, T., Tan, N., Boshuizen, H., Gick, R., Isba, R., Mann, K., Scherpbier, A., Spencer, J., & Timmins, E. (2014). How and what do medical students learn in clerkships? Experience based learning (ExBL). *Advances in Health Sciences Education,**19*(5), 721–749. 10.1007/s10459-014-9501-0 24638146 10.1007/s10459-014-9501-0

[CR18] Dyrbye, L., & Shanafelt, T. (2016). A narrative review on burnout experienced by medical students and residents. *Medical Education,**50*(1), 132–149. 10.1111/medu.12927 26695473 10.1111/medu.12927

[CR19] Edmondson, A. (1999). Psychological safety and learning behavior in work teams. *Administrative Science Quarterly,**44*(2), 350–383. 10.2307/2666999

[CR20] Frank, A. W. (2010). *Letting stories breathe: A socio-narratology*. University of Chicago Press.

[CR21] Frank, A. W. (2012). Practicing dialogical narrative analysis. In J. A. Holstein & J. F. Gubrium (Eds.), *Varieties of narrative analysis* (pp. 33–52). SAGE Publications.

[CR22] Frankl, V. E. (1985). *Man’s search for meaning*. Simon and Schuster.

[CR23] Gaufberg, E. H., Batalden, M., Sands, R., & Bell, S. K. (2010). The hidden curriculum: What can we learn from third-year medical student narrative reflections? *Academic Medicine,**85*(11), 1709–1716. 10.1097/acm.0b013e3181f57899 20881818 10.1097/ACM.0b013e3181f57899

[CR52] Gibson, D. M., Dollarhide, C. T., & Moss, J. M. (2014). Professional identity development: A grounded theory of transformational tasks of counselors. *Journal of Counseling & Development,**92*(1), 3–12. 10.1002/j.1556-6978.2010.tb00106.x

[CR24] Graves, R. (2017). *The Greek Myths—The complete and definitive edition*. Penguin Books Ltd.

[CR25] Gubrium, J. F., & Holstein, J. A. (2009). *Analyzing narrative reality*. SAGE Publications.

[CR26] Haque, O. S., & Waytz, A. (2012). Dehumanization in medicine: Causes, solutions, and functions. *Perspectives on Psychological Science,**7*(2), 176–186. 10.1177/1745691611429706 26168442 10.1177/1745691611429706

[CR27] Holquist, M. (2003). *Dialogism: Bakhtin and his world*. Routledge.

[CR28] Jarvis-Selinger, S., Pratt, D. D., & Regehr, G. (2012). Competency is not enough: Integrating identity formation into the medical education discourse. *Academic Medicine,**87*(9), 1185–1190. 10.1097/acm.0b013e3182604968 22836834 10.1097/ACM.0b013e3182604968

[CR29] Johna, S., & Rahman, S. (2011). Humanity before science: Narrative medicine, clinical practice, and medical education. *The Permanente Journal,**15*(4), 92. 10.7812/tpp/11-111 10.7812/tpp/11-111 PMC3267572 22319427

[CR30] Josselson, R. (2011). “Bet you think this song is about you”: Whose narrative is it in narrative research? *Narrative Matters,**1*(1), 33–51.

[CR31] Jung, C. G. (1951). Fundamental questions of psychotherapy. In C. G. Jung (Ed.), *The collected works of CG Jung* (pp. 111–125). Princeton University Press.

[CR32] Kerasidou, A., & Horn, R. (2016). Making space for empathy: Supporting doctors in the emotional labour of clinical care. *BMC Medical Ethics,**17*(1), 1–5. 10.1186/s12910-016-0091-7 26818248 10.1186/s12910-016-0091-7 PMC4728886

[CR33] Kirmayer, L. J., Gómez-Carrillo, A., Sukhanova, E., & Garrido, E. (2023). Narrative medicine. In J. E. Mezzich, W. J. Appleyard, P. Glare, J. Snaedal, & C. R. Wilson (Eds.), *Person centered medicine* (pp. 235–255). Springer Cham.

[CR34] Kleinman, A. (2020). *The illness narratives: Suffering, healing, and the human condition*. Basic Books. 10.1097/ACM.0000000000001864 28952997

[CR35] Konopasky, A., Varpio, L., & Stalmeijer, R. E. (2021). The potential of narrative analysis for HPE research: Highlighting five analytic lenses. *Medical Education,**55*(12), 1369–1375. 10.1111/medu.14597 34291492 10.1111/medu.14597

[CR36] Krasner, M. S., Epstein, R. M., Beckman, H., Suchman, A. L., Chapman, B., Mooney, C. J., & Quill, T. E. (2009). Association of an educational program in mindful communication with burnout, empathy, and attitudes among primary care physicians. *JAMA,**302*(12), 1284–1293. 10.1001/jama.2009.1384 19773563 10.1001/jama.2009.1384

[CR37] Laine, C., & Davidoff, F. (1996). Patient-centered medicine: A professional evolution. *JAMA,**275*(2), 152–156. 10.1001/jama.1996.03530260066035 8531314

[CR38] Lauckner, H., Doucet, S., & Wells, S. (2012). Patients as educators: The challenges and benefits of sharing experiences with students. *Medical Education,**46*(10), 992–1000. 10.1111/j.1365-2923.2012.04356.x 22989133 10.1111/j.1365-2923.2012.04356.x

[CR39] Launer, J., & Wohlmann, A. (2023). Narrative medicine, narrative practice, and the creation of meaning. *The Lancet,**401*(10371), 98–99. 10.1016/s0140-6736(23)00017-x 10.1016/S0140-6736(23)00017-X 36641204

[CR40] Lee, R. M., & Renzetti, C. M. (1990). The problems of researching sensitive topics: An overview and introduction. *American Behavioral Scientist,**33*(5), 510–528. 10.1177/0002764290033005002

[CR41] Lefkowitz, A., Vizza, J., & Kuper, A. (2022). Patients as experts in the illness experience: Implications for the ethics of patient involvement in health professions education. *Journal of Evaluation in Clinical Practice,**28*(5), 794–800. 10.1111/jep.13672 35274414 10.1111/jep.13672

[CR42] Liao, K.-C., Ajjawi, R., Peng, C.-H., Jenq, C.-C., & Monrouxe, L. V. (2023). Striving to thrive or striving to survive: Professional identity constructions of medical trainees in clinical assessment activities. *Medical Education*. 10.1111/medu.15152 10.1111/medu.15152 37394612

[CR43] Lim, J. Y., Ong, S. Y. K., Ng, C. Y. H., Chan, K. L. E., Wu, S. Y. E. A., So, W. Z., Tey, G. J. C., Lam, Y. X., Gao, N. L. X., & Lim, Y. X. (2023). A systematic scoping review of reflective writing in medical education. *BMC Medical Education,**23*(1), 12. 10.1186/s12909-022-03924-4 36624494 10.1186/s12909-022-03924-4 PMC9830881

[CR44] Lönn, A., Weurlander, M., Seeberger, A., Hult, H., Thornberg, R., & Wernerson, A. (2023). The impact of emotionally challenging situations on medical students’ professional identity formation. *Advances in Health Sciences Education*. 10.1007/s10459-023-10229-8 10.1007/s10459-023-10229-8 PMC10184105 37184676

[CR45] McAdams, D. P. (2011). Narrative identity. In S. J. Schwartz, K. Luyckx, & V. L. Vignoles (Eds.), *Handbook of Identity Theory and Research* (pp. 99–115). Springer.

[CR46] McAdams, D. P., & McLean, K. C. (2013). Narrative identity. *Current Directions in Psychological Science,**22*(3), 233–238. 10.1177/0963721413475622

[CR47] Merchant, J. (2012). *Shamans and Analysts: New Insights on the Wounded Healer*. Routledge.

[CR48] Merton, R. K., Reader, G., & Kendall, P. L. (1957). *The Student Physician: Introductory Studies in the Sociology of Medical Education*. Harvard University Press.

[CR49] Milota, M. M., van Thiel, G. J., & van Delden, J. J. (2019). Narrative medicine as a medical education tool: A systematic review. *Medical Teacher,**41*(7), 802–810. 10.1080/0142159x.2019.1584274 30983460 10.1080/0142159X.2019.1584274

[CR50] Monrouxe, L. V. (2010). Identity, identification and medical education: Why should we care? *Medical Education,**44*(1), 40–49. 10.1111/j.1365-2923.2009.03440.x 20078755 10.1111/j.1365-2923.2009.03440.x

[CR51] Monrouxe, L. V., Rees, C. E., & Hu, W. (2011). Differences in medical students’ explicit discourses of professionalism: Acting, representing, becoming. *Medical Education,**45*(6), 585–602. 10.1111/j.1365-2923.2010.03878.x 21564198 10.1111/j.1365-2923.2010.03878.x

[CR53] Nouwen, H. J. (1979). *The Wounded Healer: Ministry in Contemporary Society*. Image.

[CR54] Passi, V., Johnson, S., Peile, E., Wright, S., Hafferty, F., & Johnson, N. (2013). Doctor role modelling in medical education: BEME Guide No. 27. *Medical Teacher,**35*(9), e1422–e1436. 10.3109/0142159x.2013.806982 23826717 10.3109/0142159X.2013.806982

[CR55] Reeve, J. (2010). Interpretive medicine: Supporting generalism in a changing primary care world. *Occasional Paper (royal College of General Practitioners),**88*, 1–20. PMC3259801 21805819

[CR56] Roebotham, T., Hawthornthwaite, L., Lee, L., & Lingard, L. A. (2018). Beyond catharsis: The nuanced emotion of patient storytellers in an educational role. *Medical Education,**52*(5), 526–535. 10.1111/medu.13510 29430729 10.1111/medu.13510

[CR57] Santana, M. J., Manalili, K., Jolley, R. J., Zelinsky, S., Quan, H., & Lu, M. (2018). How to practice person-centred care: A conceptual framework. *Health Expectations,**21*(2), 429–440. 10.1111/hex.12640 29151269 10.1111/hex.12640 PMC5867327

[CR58] Sarraf-Yazdi, S., Teo, Y. N., How, A. E. H., Teo, Y. H., Goh, S., Kow, C. S., Lam, W. Y., Wong, R. S. M., Ghazali, H. Z. B., & Lauw, S.-K. (2021). A scoping review of professional identity formation in undergraduate medical education. *Journal of General Internal Medicine,**36*(11), 3511–3521. 10.1007/s11606-021-07024-9 34406582 10.1007/s11606-021-07024-9 PMC8606368

[CR59] Schei, E. (2015). *Lytt: Legerolle og Kommunikasjon*. Fagbokforlaget.

[CR60] Schei, E., Knoop, H. S., Gismervik, M. N., Mylopoulos, M., & Boudreau, J. D. (2019). Stretching the comfort zone: Using early clinical contact to influence professional identity formation in medical students. *Journal of Medical Education and Curricular Development,**6*, 2382120519843875. 10.1177/2382120519843875 31065588 10.1177/2382120519843875 PMC6487753

[CR61] Shapiro, J. (2008). Walking a mile in their patients’ shoes: empathy and othering in medical students’ education. *Philosophy, Ethics, and Humanities in Medicine,**3*, 10. 10.1186/1747-5341-3-10 10.1186/1747-5341-3-10 PMC2278157 18336719

[CR62] Shapiro, J. (2011). Perspective: Does medical education promote professional alexithymia? A call for attending to the. *Academic Medicine,**86*(3), 326–332. 10.1097/acm.0b013e3182088833 21248595 10.1097/ACM.0b013e3182088833

[CR63] Shapiro, J. (2013). The feeling physician: Educating the emotions in medical training. *European Journal for Person Centered Healthcare,**1*(2), 310–316. 10.5750/ejpch.v1i2.664

[CR64] Slavich, G. M., & Zimbardo, P. G. (2012). Transformational teaching: Theoretical underpinnings, basic principles, and core methods. *Educational Psychology Review,**24*(4), 569–608. 10.1007/s10648-012-9199-6 23162369 10.1007/s10648-012-9199-6 PMC3498956

[CR65] Stewart, M., Brown, J. B., Weston, W., McWhinney, I. R., McWilliam, C. L., & Freeman, T. (2013). *Patient-centered medicine: Transforming the clinical method*. CRC Press.

[CR66] Toufan, N., Omid, A., & Haghani, F. (2023). The double-edged sword of emotions in medical education: A scoping review. *Journal of Education and Health Promotion*. 10.4103/jehp.jehp_644_21 10.4103/jehp.jehp_644_21 PMC10127485 37113412

[CR67] Warmington, S., & McColl, G. (2017). Medical student stories of participation in patient care-related activities: The construction of relational identity. *Advances in Health Sciences Education,**22*, 147–163. 10.1007/s10459-016-9689-2 27235124 10.1007/s10459-016-9689-2

[CR68] Watling, C. J., & Ginsburg, S. (2019). Assessment, feedback and the alchemy of learning. *Medical Education,**53*(1), 76–85. 10.1111/medu.13645 30073692 10.1111/medu.13645

[CR69] Weiner, S. J., & Schwartz, A. (2016). Contextual errors in medical decision making: Overlooked and understudied. *Academic Medicine,**91*(5), 657–662. 10.1097/acm.0000000000001017 26630603 10.1097/ACM.0000000000001017

[CR70] Wenger, E. (1998). *Communities of Practice: Learning, Meaning, and Identity*. Cambridge University Press.

[CR71] Whelan, B., Hjörleifsson, S., & Schei, E. (2021). Shame in medical clerkship:“You just feel like dirt under someone’s shoe.” *Perspectives on Medical Education,**10*(5), 265–271. 10.1007/s40037-021-00665-w 33950359 10.1007/s40037-021-00665-w PMC8505567

[CR72] Wilcox, M. V., Orlando, M. S., Rand, C. S., Record, J., Christmas, C., Ziegelstein, R. C., & Hanyok, L. A. (2017). Medical students’ perceptions of the patient-centredness of the learning environment. *Perspectives on Medical Education,**6*, 44–50. 10.1007/s40037-016-0317-x 27987074 10.1007/s40037-016-0317-x PMC5285277

[CR73] Wilt, J. A., Thomas, S., & McAdams, D. P. (2019). Authenticity and inauthenticity in narrative identity. *Heliyon*. 10.1016/j.heliyon.2019.e02178 10.1016/j.heliyon.2019.e02178 PMC6676168 31388595

[CR74] Wong, A., & Trollope-Kumar, K. (2014). Reflections: An inquiry into medical students’ professional identity formation. *Medical Education,**48*(5), 489–501. 10.1111/medu.12382 24712934 10.1111/medu.12382

[CR75] Zerubavel, N., & Wright, M. O. D. (2012). The dilemma of the wounded healer. *Psychotherapy,**49*(4), 482. 10.1037/a0027824 22962968 10.1037/a0027824

